# Supplementary material for: Continuous addition of progenitors forms the cardiac ventricle in zebrafish
Source: Nat Commun. 2018 May 21;9:2001. doi: 10.1038/s41467-018-04402-6 (PMC5962599; doi:10.1038/s41467-018-04402-6)
Supplement: Supplementary file 1 — Supplementary Information [file 41467_2018_4402_MOESM1_ESM.pdf]

Supplementary Information

Continuous addition of progenitors forms the cardiac ventricle in zebrafish

Felker et al.

Supplementary Figures 1-7

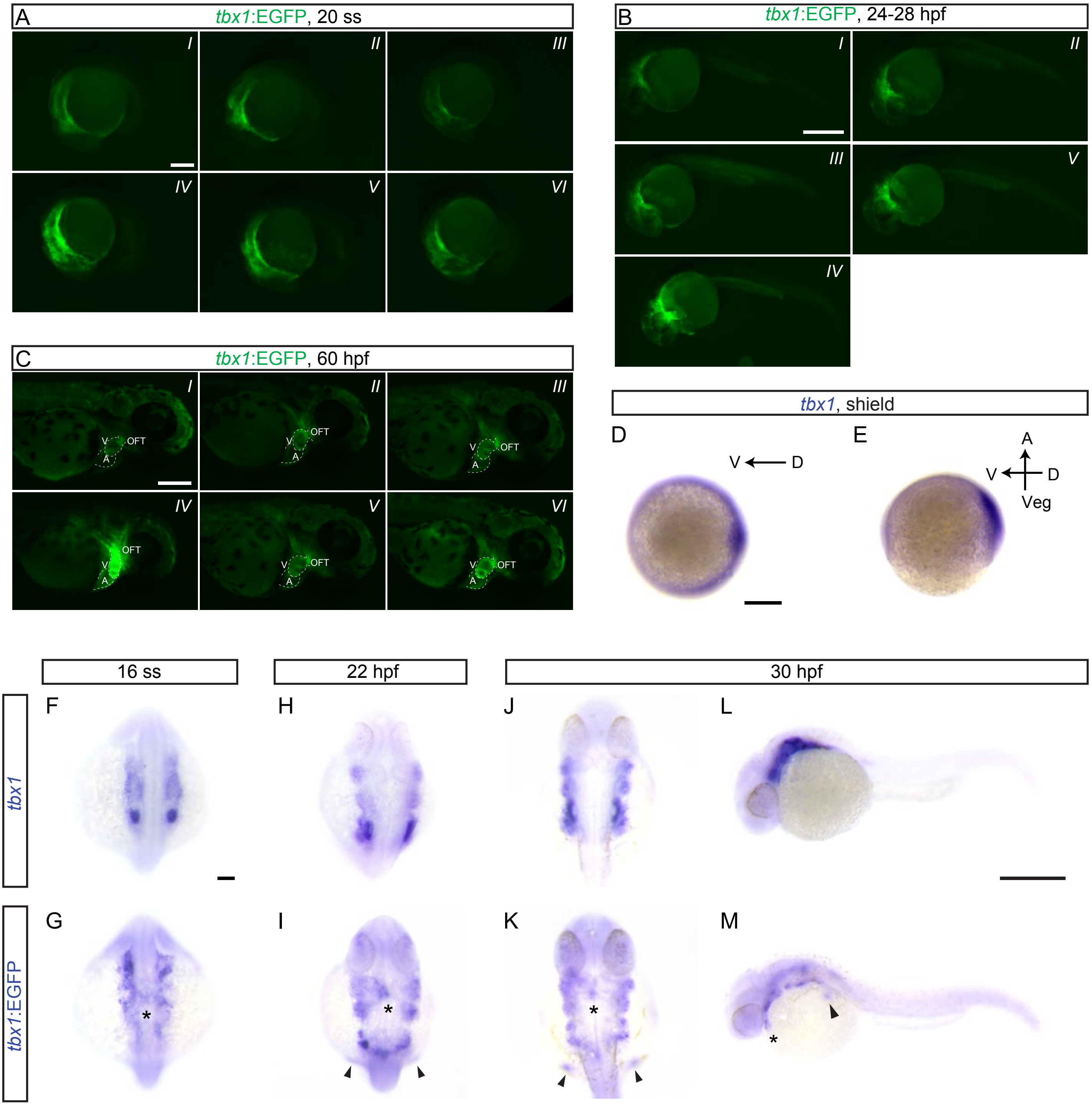

Supplementary Figure 1: *tbx1* reporter- and endogenous expression in different transgenic lines

(**A,B**) Anterior expression in different transgenic *tbx1:EGFP* lines at 20 ss (**A**) and 24-28 hpf (**B**); lateral views, anterior to the left. Expression in all lines (labeled with Roman numerals *I-VI*) is confined to the anterior CPF with some minor unspecific signals in the notochord or scattered posterior skeletal muscles (see Supplementary Table 1). (**C**) Cardiac *tbx1*:EGFP expression in all lines is strongest in the ventricle (V) and outflow tract (OFT) and weaker in the atrium (A) at 60 hpf; lateral view of the right side of the embryo due to better visibility of the outflow tract, anterior to the right; dashed outline indicates the heart. (**D,E**) Endogenous *tbx1* expression confines to a dorsal domain during gastrulation; (**D**) animal view, (**E**) lateral view; V: ventral, D: dorsal, A: animal, Veg: vegetal. (**F-K**) By mRNA ISH, *tbx1:EGFP* and *tbx1* expression is detected in anterior pharyngeal precursors from mid-somitogenesis to heart tube stages (16 ss to 30 hpf). Additionally, *tbx1* reporter expression marks the migrating heart field at the midline (asterisk) and emerging fin bud precursors (arrowheads); dorsal views, anterior to the top. (**L,M**) *tbx1:EGFP* and *tbx1* are confined to anterior domains with no expression in posterior structures; asterisks heart tube in *tbx1*:EGFP, arrowhead fin bud precursors; lateral views, anterior to the left. Scale bars 100 µm (**F-K**), 250 µm (**A,C**), 500 µm (**B,L,M**).

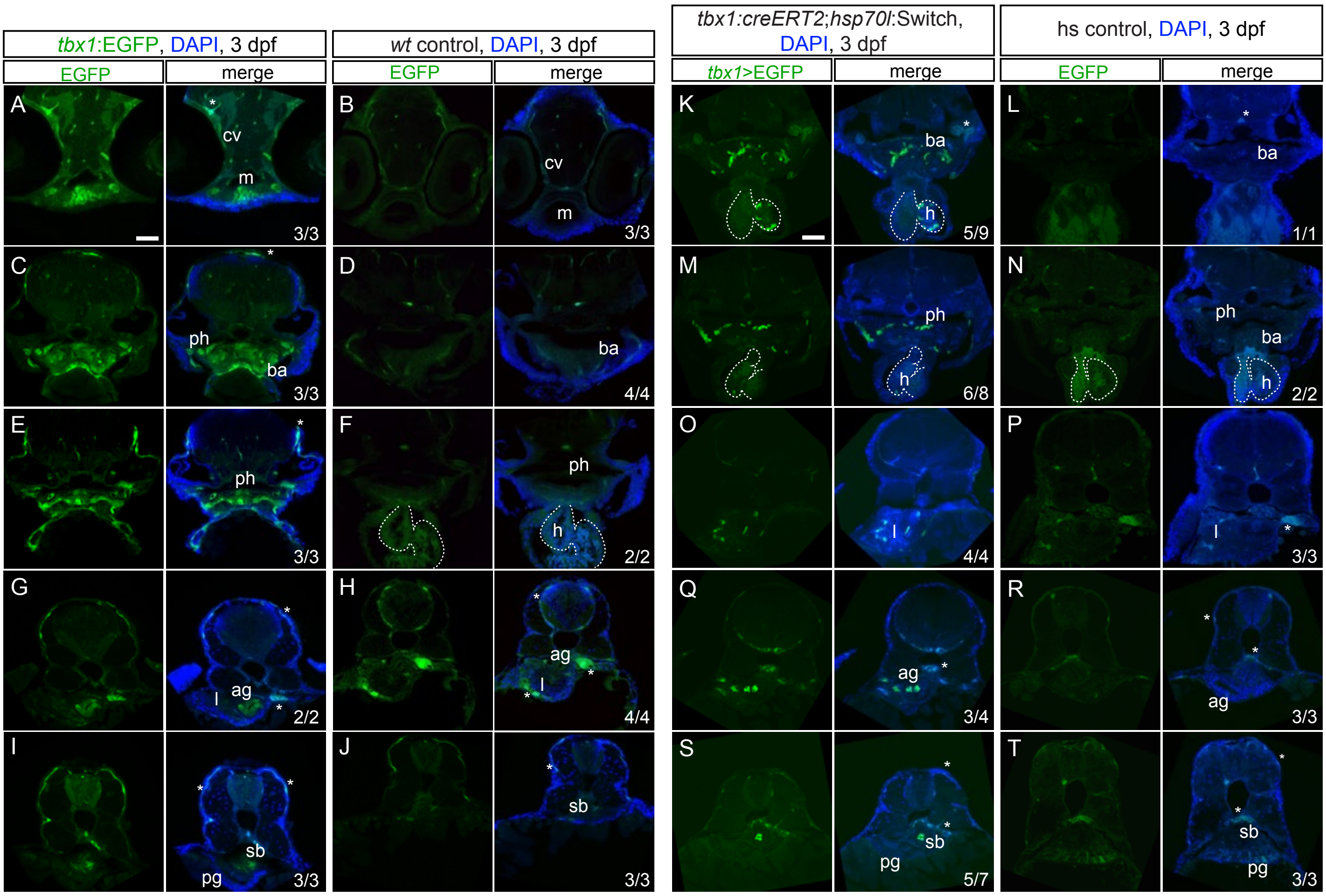

Supplementary Figure 2: *tbx1* reporter expression and CreERT2/*lox*-mediated lineage tracing

(**A-T**) Transverse agarose sections of 3 dpf transgenic or control embryos aligned top to bottom from anterior to posterior with endogenous EGFP reporter fluorescence (green) and nuclear stain (DAPI, blue); top-down confocal Z-sections; dorsal to the top. Numbers in the bottom right corner indicate the number of individual embryos with expression in represented structures at a similar position along the anterior-posterior axis out of the total number of embryos analyzed for the respective region. Asterisks indicate autofluorescent signals in skin and blood that can also be observed in control sections. (**A,B**) In the anterior-most sections, *tbx1:EGFP* reporter expression is confined to neural crest-derived mandibular cartilage (m) and ALPM-derived cranial vasculature (cv). (**C-F**) Further posterior branchial arches (ba, containing mesodermal, endodermal and neural crest lineages) and the endodermal pharynx (ph) express *tbx1*:EGFP. (**G-J**) *tbx1*:EGFP reporter expression can be also found in the endoderm-derived swim bladder (sb) and in the anterior gut (ag), and weakly in the liver (l) but not in the posterior gut segments (pg) at the height of the swim bladder, suggesting absence of *tbx1* reporter activity in more posterior segments. (**K-T**) Akin to *tbx1*:EGFP reporter expression, *tbx1:creERT2;hsp70l*:Switch-mediated lineage tracing throughout gastrulation (4-OHT induction between shield-bud, *tbx1*>EGFP) marks the branchial arches (ba), pharynx (ph), anterior gut (ag), liver (l), and swim bladder (sb). As observed by live imaging of lineage-traced embryos (Figure 2S-ZZ), ventricular myocardium of the heart (h) is also marked. The posterior gut was not labelled in 5/7 embryos, supporting loss of enhancer activity in more posterior endodermal precursors. (**A-T**) No organ-specific EGFP signal or unspecific switching activity can be observed in non-transgenic (wildtype, *wt*) or non-4-OHT-induced *tbx1*:creERT2;*hsp70l*:Switch heatshock (hs) controls, but autofluorescent signal in the skin and blood can be observed in controls and throughout all sections (asterisks **A-T**). Scale bars 500 µm.

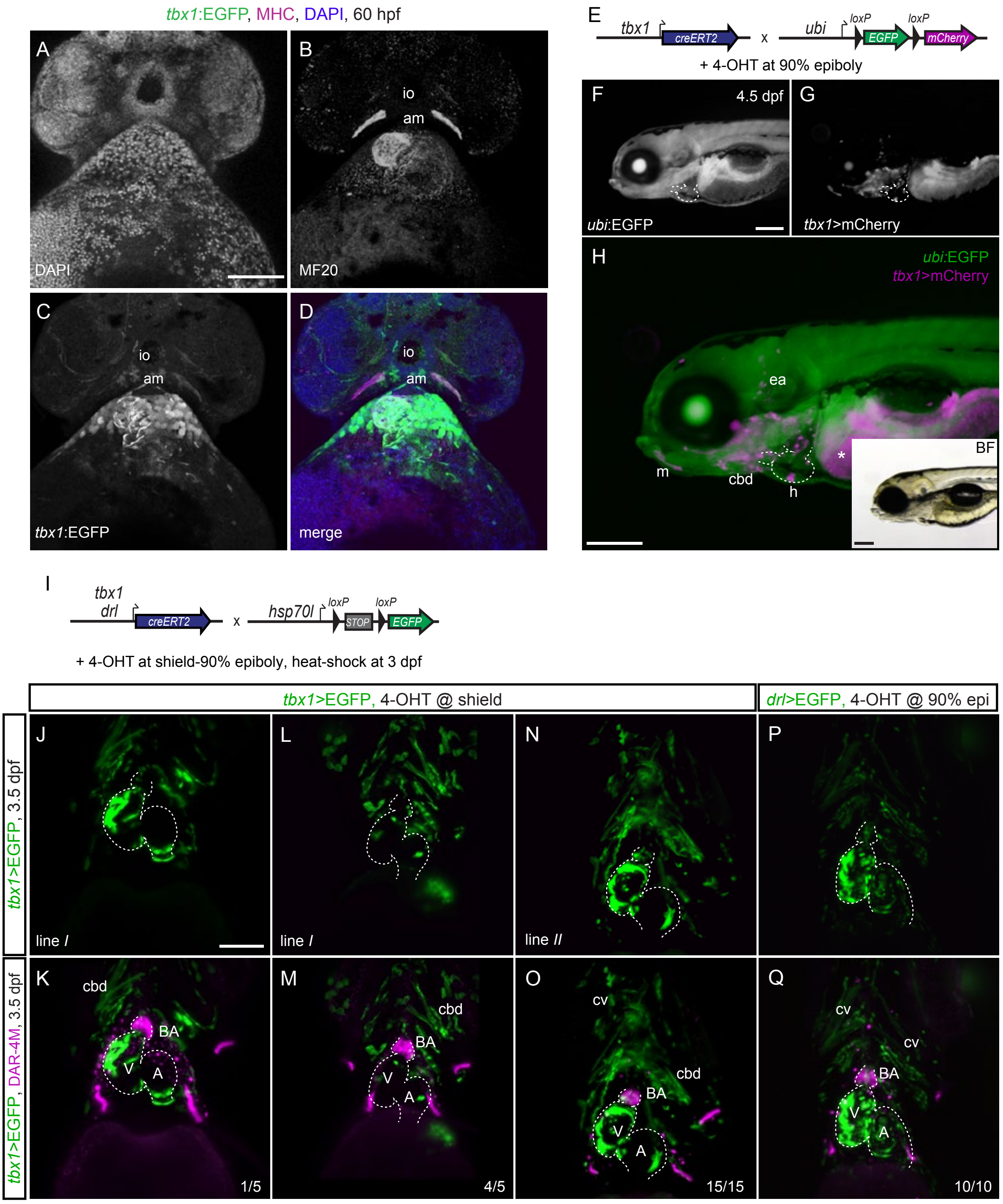

Supplementary Figure 3: *tbx1* reporter expression and lineage tracing in cardiopharyngeal tissues

(**A-D**) Maximum intensity projections of fixed whole-mount immunofluorescence from 60 hpf *tbx1*:EGFP embryos counterstained with anti-EGFP and anti-MHC; ventral view, anterior to the top. *tbx1*:EGFP-positive cranio-branchial derivatives observed through lineage tracing and reporter expression in previous experiments include the cranial muscles inferior oblique (io) and adductor mandibulae (am). (**E**) *tbx1:creERT2* transgenics were crossed to *ubi:Switch* and induced with 4-OHT at the end of gastrulation (90% epiboly). *ubi:Switch* (*ubi:loxP-EGFP-loxP-mCherry*) switches from EGFP to mCherry expression in all cells expressing the *creERT2* transgene at the time of 4-OHT induction. (**F-H**) mCherry-expressing *tbx1:creERT2* derivatives (*tbx1*>mCherry) at 4.5 dpf can be found in the heart (h, outlined), cranio-branchial derivatives (cbd), mandibular cartilage (m), and in the ear (ea). The inlet in (**H**) depicts the brightfield image of **F-H**; lateral view, anterior to the left. (**I**) *tbx1:creERT2* or *drl:creERT2* transgenics were crossed to the ubiquitous *hsp70l:Switch loxP* tracer line, 4-OHT-induced at shied stage or 90% epiboly, respectively, and heat-shocked at 3 dpf. (**J-Q**) Maximum intensity projections from SPIM-imaged 3.5 dpf embryo; ventral views, anterior to the top; dashed outlines indicate the heart. (**J-O**) *tbx1*:CreERT2-mediated lineage tracing was induced with 4-OHT at shield stage to capture the earliest derivatives of *tbx1* reporter-expressing progenitors and compared in two independent transgenic lines (line *I* and *II*). We reproducibly found *tbx1* lineage-traced cells (*tbx1*>EGFP) in ventricular (V) myocardium, atrial (A) myocardium at the inflow tract, and in the DAR-4M-marked bulbus arteriosus (BA). We observed more consistent switching efficiencies in the heart with *tbx1:creERT2* line *II*. Additionally, cranio-branchial derivatives (cbd) and cranial vessels (cv) are readily marked by *tbx1* lineage tracing. (**P,Q**) *drl:creERT2*-mediated lineage tracing (*drl*>EGFP) was initiated at 90% epiboly to label LPM derivatives that can be found in all cardiac structures (V, A, BA) and cranial vasculature (cv). Scale bars 100 μm (**A-D**,**J-Q**), 250 μm (**F-H**).

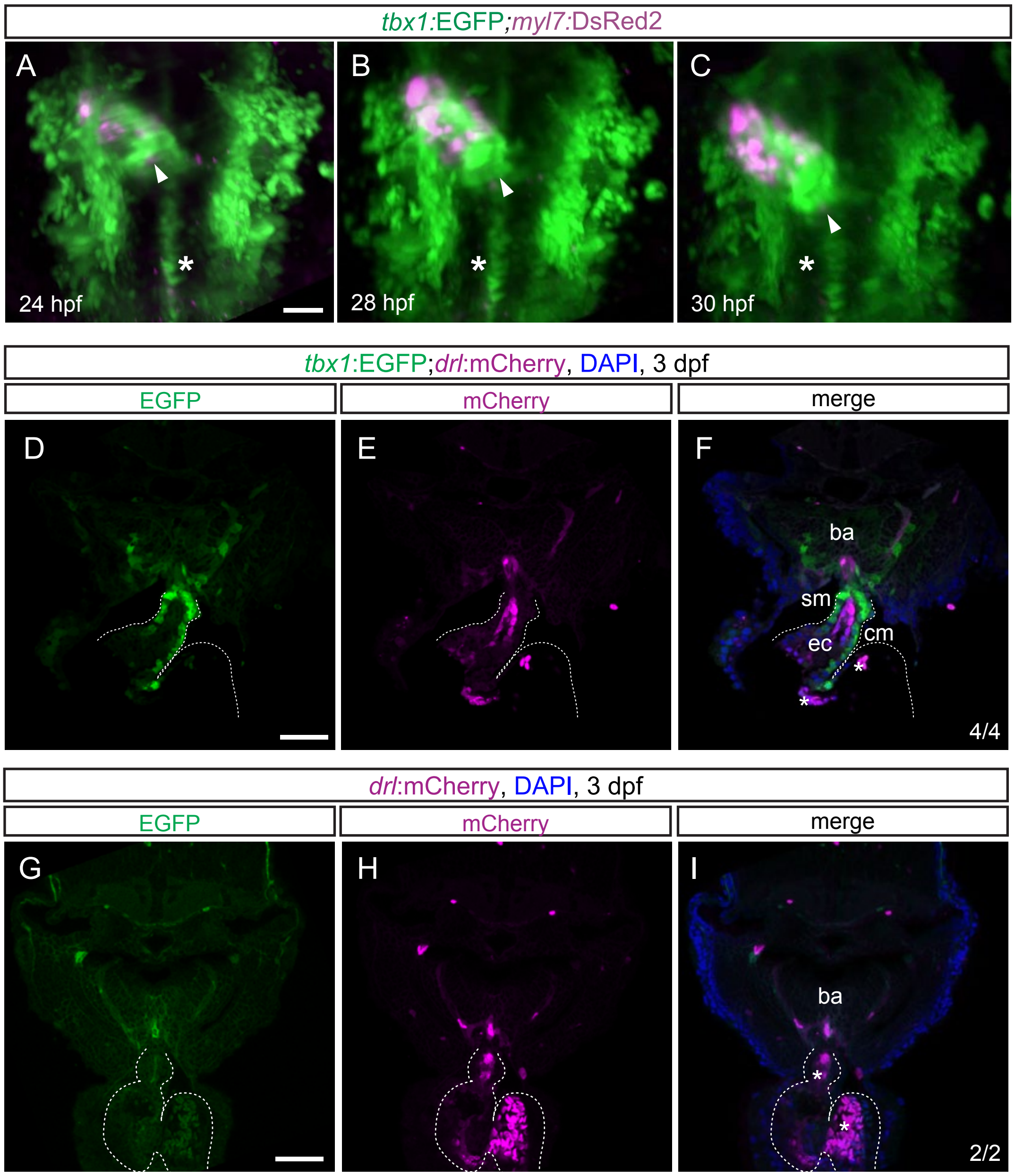

Supplementary Figure 4: *tbx1* reporter-expression at the base of the linear heart tube and in the BA
(**A-C**) Maximum intensity projections of representative stages from SPIM-imaged *tbx1:EGFP*;*myl7:DsRed2* double-transgenic embryos (n=2), dorsal views, anterior to the top. Imaging was initiated at 18 hpf and heart development was followed up to 30 hpf. *tbx1:*EGFP-only cells were observed at the base of the *myl7:*DsRed2-positive linear heart tube at all stages imaged (arrow heads). Imaging was performed on *tbx1:EGFP line IV* embryos; note the strong notochord expression (asterisk) not observed in *line I* (see also Supplementary Figure 1 and Supplementary Table 1). (**D-I**) Transverse agarose sections of *tbx1:EGFP*;*drl:mCherry* (**D-F**) or *drl:mCherry* (**G-I**) transgenic embryos with endogenous EGFP (green) and mCherry (magenta) reporter fluorescence and nuclear stain (DAPI, blue); top-down confocal z-sections; dorsal to the top; dashed outline marks the heart. *drl*:mCherry is expressed in endothelial cells (ec) and blood (asterisks), *tbx1*:EGFP is specific to the smooth muscle layer of the bulbus arteriosus (sm). *tbx1* reporter expression can be also detected in the myocardium of the inner curvature (cm) and in the branchial arches (ba). Scale bars 50 µm.

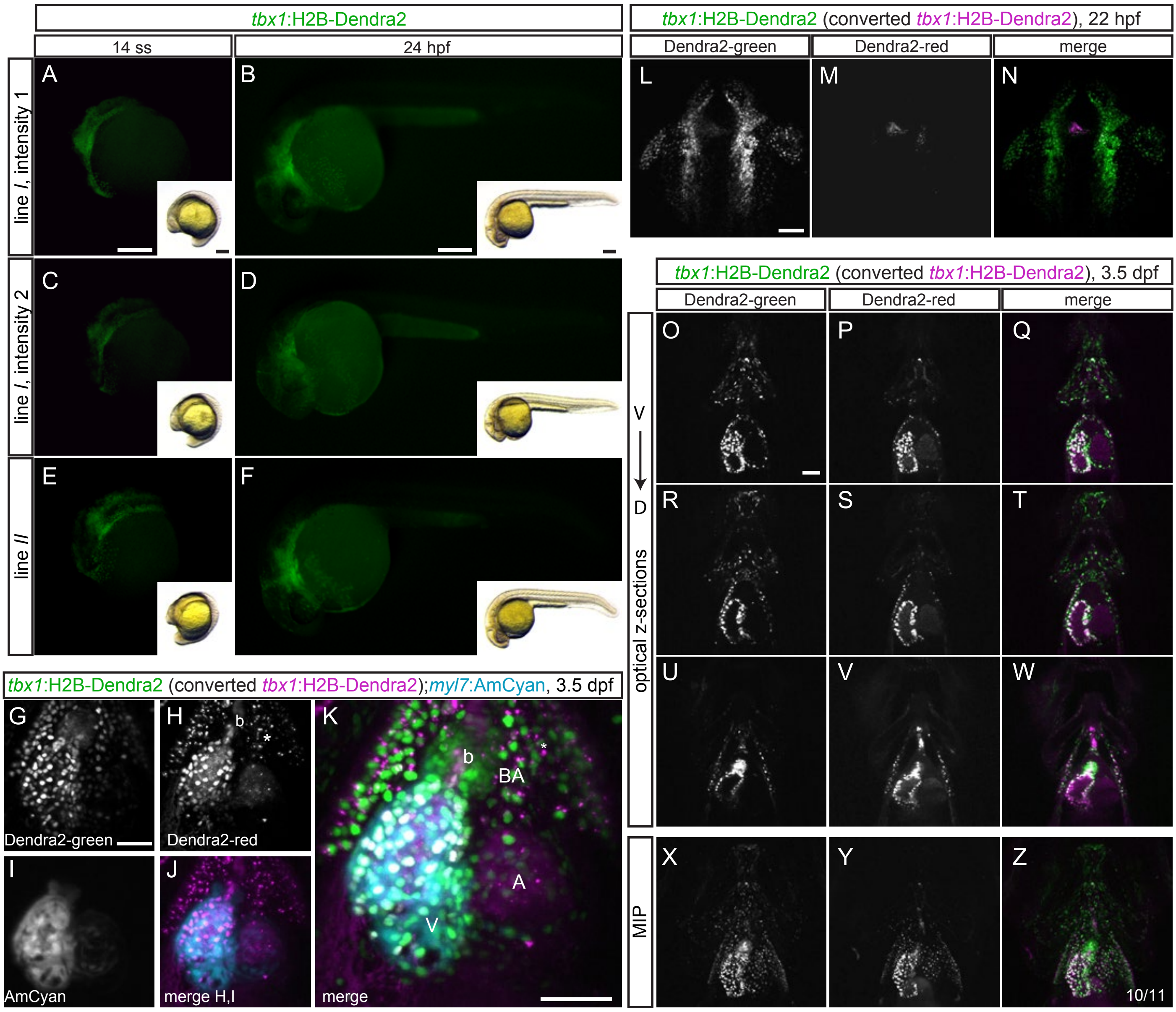

Supplementary Figure 5: Myocardium in all ventricular segments derives from the *tbx1* reporter sheath
(**A-F**) Anterior expression in two independent transgenic *tbx1:Dendra2* lines at 14 ss and 24 hpf; lateral views, anterior to the left; insets depict brightfield images of the respective fluorescent images. We observed the same reporter expression patterns as in the *tbx1:EGFP* lines (see Supplementary Fig. 1) in both lines. In line *I*, we noted germline transmission in more than 50% of F2 generation embryos likely due to different insertions still present in the line, resulting in varying fluorescent intensities (see **A-D**). (**G-K**) Maximum intensity projections of the heart shown in Figure 5C,D with *myl7*:AmCyan demonstrating that photoconverted cells from the *tbx1* reporter-expressing sheath at the base of the forming heart tube at 22 hpf contribute to ventricular myocardium at 3.5 dpf; ventral view, anterior to the top. (**L-N**) A second representative *tbx1*:Dendra2 embryo converted at the base of the forming heart tube at 22 hpf is shown; maximum intensity projection, dorsal view, anterior to the top. (**O-Z**) Live SPIM-imaging of the same embryo depicted in (**G**) at 3.5 dpf; ventral view, anterior to the top. Optical z-sections from ventral (**O-Q**), medial (**R-T**) and dorsal (**U-W**) regions as well as the maximum intensity projection (**X-Z**) show that regions of the ventricular myocardium, including most distal segments, but not the bulbus arteriosus, are contributed from the photoconverted sheath at 22 hpf. Dendra2-red in all or only in the distal-most ventricular myocardium (as depicted in **G-K**) was observed in 10/11 embryos with *tbx1* reporter-expressing sheath conversion at 22 hpf. In 1/11 embryos, no Dendra2-red cells could be found in the entire anterior region of the embryo. Scale bars 50 µm (**G-Z**), 250 µm (**A-F**).

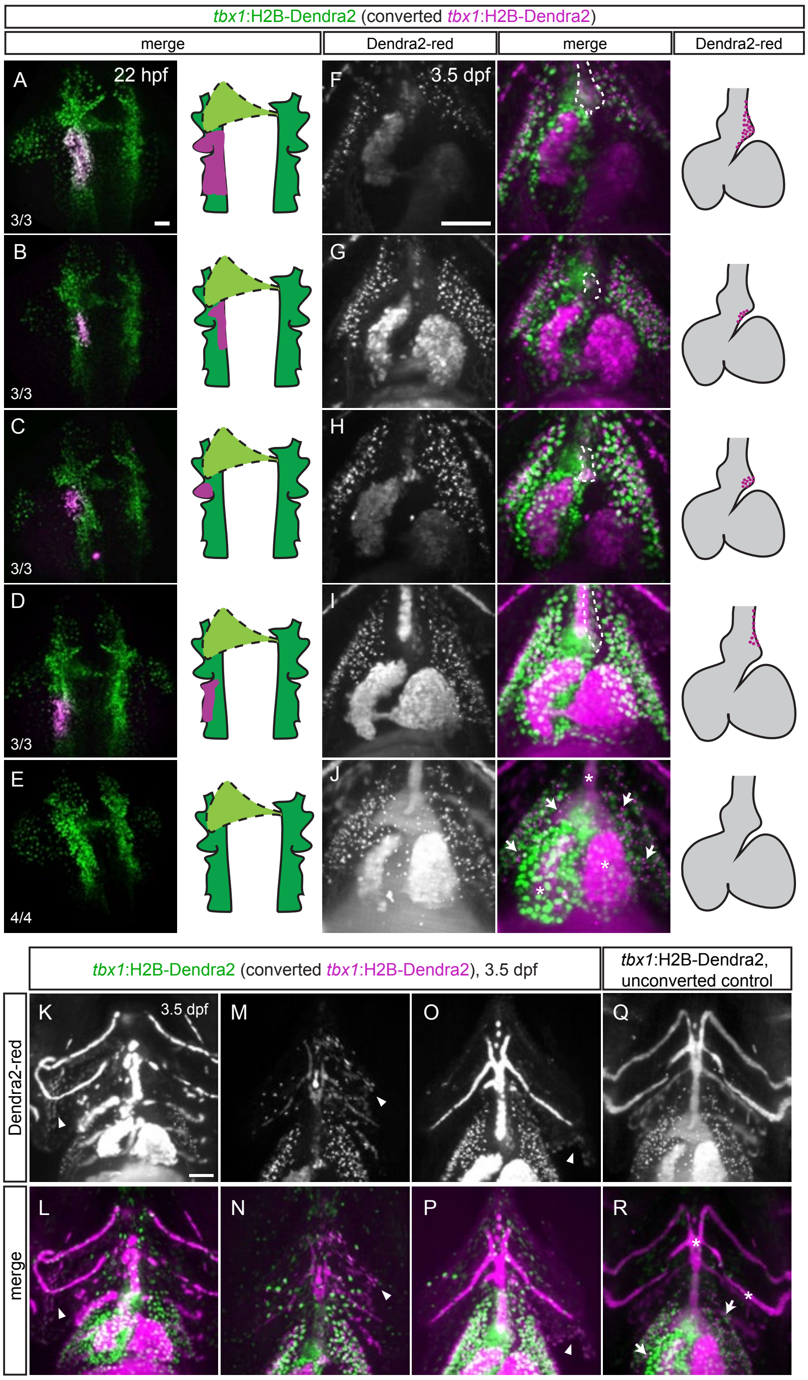

Supplementary Figure 6: Different regions of the pharyngeal LPM contribute to distinct parts of the BA and craniofacial structures

(**A-E**) Maximum intensity projections and schematics of representative photoconverted and control *tbx1*:Dendra2 embryos; dorsal views, anterior to the top, schematics depicting structures and converted area. At 22 hpf, *tbx1*:Dendra2-expressing embryos were illuminated with UV light in a confined region of interest to convert Dendra2-green to Dendra2-red in specific *tbx1* reporter-expressing domains. (**F-J**) Maximum intensity projections and graphical representations of hearts of *tbx1*:Dendra2 embryos photoconverted in (**A-E**) at 3.5 dpf with marked contribution to the heart by photoconverted cells; ventral views, anterior to the top. (**A,F**) Photoconversion of a broad area in the *tbx1*:Dendra2-positive pharyngeal LPM posterior to and on the left of the cardiac cone marks the left side of the BA (dotted outline). (**B,G**) The most medial region of the area converted in (**A**) contributes to the most proximal part of the BA suggesting that these progenitors migrate and add the earliest to the heart. (**C,H**) A lateral and anterior region to (**B**) contributes to the medial part of the BA. (**D,I**) The most posterior and lateral region makes the distal part of the BA and contributes to cells of the ascending aorta. (**E,J**) Autofluorescent signal in the red channel can be picked up in blood (asterisks) and on top of the pericardium (arrows) as observed in non-photoconverted control embryos. (**K-R**) Maximum intensity projections of photoconverted *tbx1*:Dendra2 embryos at 3.5 dpf; ventral views, anterior to the top. Photoconverted regions as in (**B-E**), (**M-P**) depict the same embryos as shown in (**C**) and (**D**), respectively; (**K,L**) shows an embryo that was photoconverted in an equivalent but right-hand sided region as shown in (**B**). Progenitors of different parts of the BA derive from the same areas as different parts of craniofacial muscles and cartilage. (**Q,R**) Maximum intensity projections of the unconverted *tbx1*:Dendra2 embryo as shown in (**E**); ventral view, anterior to the top. Red signal from blood (asterisks) and on top of the pericardium (arrows) were observed in unconverted Dendra2 embryos imaged at 3.5 dpf using lightsheet microscopy, suggesting unspecific auto-fluorescence in these structures picked up at the imaging conditions used for Dendra2 experiments. Scale bars 50 µm.

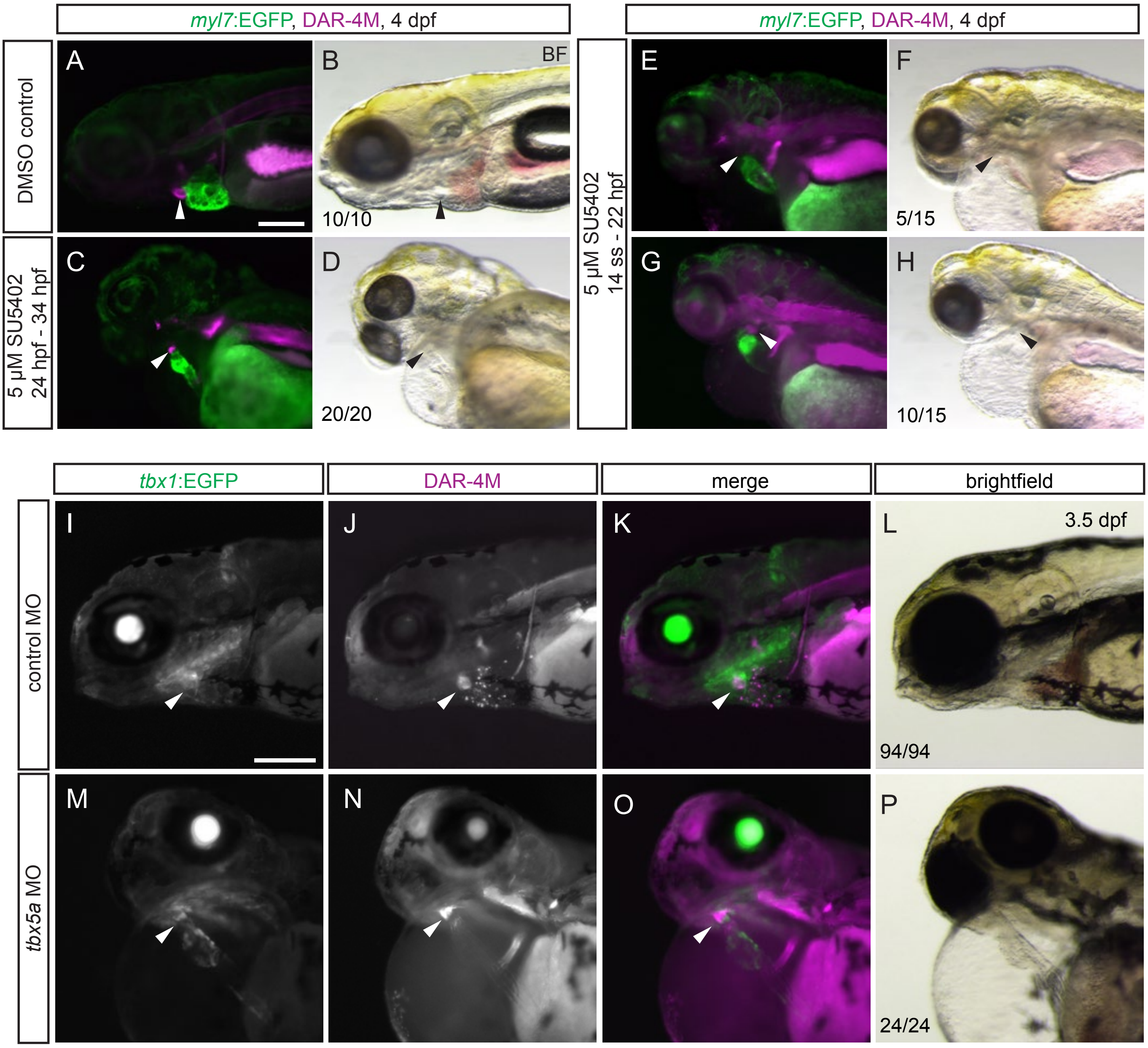

Supplementary Figure 7: Aberrant formation of the DAR-4M-stained BA upon FGF signaling perturbation
(**A-H**) *myl7*:EGFP, DAR-4M-stained embryos treated with DMSO or 5μM SU5402 during (14 ss- 22hpf) or after (24 hpf-34 hpf) heart tube formation; lateral views, anterior to the left. Absent BA formation can only be observed in embryos treated with SU5402 from mid-somitogenesis to heart tube stages (arrow head **E, G** shows embryos with weaker DAR-4M signal than control in **A**), but not when signaling inhibition is initiated at 24 hpf (arrowhead **C**). (**I-P**) *tbx5a* morpholino injected embryos with the full heartstring phenotype (24 of 203 injected embryos with successful *tbx5a* knockdown assessed by fin defects (a total of 3 clutches)), all still develop a BA (n=24/24), as seen by *tbx1*:EGFP expression and DAR-4M labeling (**M-P**), excluding the possibility that the extensive edema observed in SU5402-treated embryos causes BA cells to undergo apoptosis. No heartstring phenotype or loss of the BA was observed in control morpholino injected embryos (**I-L**, n=94, 3 clutches). Scale bars 100 μm (**A-H**), 250 μm (**I-P**).

Supplementary Table 1. Comparison of *tbx1*:EGFP transgenic zebrafish lines

**Line**	**GFP Intensity (1-10)**	**% positive embryos**	**Presumed # of insertions**	**Unspecific signal**
Line I	4	50	1	none
Line II	6	50	1	notochord
Line III	2-6	>50	2	scattered skeletal muscles posterior
Line IV	9	50	1	notochord
Line V	6	50	1	notochord
Line VI	7	>50	2	none
