# Supplementary material for: Continuous addition of progenitors forms the cardiac ventricle in zebrafish
Source: Nat Commun. 2018 May 21;9:2001. doi: 10.1038/s41467-018-04402-6 (PMC5962599; doi:10.1038/s41467-018-04402-6)
Supplement: Supplementary file 3 — Description of Additional Supplementary Files [file 41467_2018_4402_MOESM3_ESM.docx]

**Description of Additional Supplementary Files**

File Name: Supplementary Movie 1

Description: Panoramic lightsheet-imaging of a *tbx1*:EGFP;*drl*:mCherry embryo. Mercator projection of early developmental stages from 8 -15 hpf, *tbx1*:EGFP is depicted in green, *drl*:mCherry in magenta, dorsal view, anterior to the left, n=3. Movie stacks were acquired every 2 min. Frame rate of the movie: 29 frames per second (fps). 1 sec in the movie corresponds approximately to 30 min in development.

File Name: Supplementary Movie 2

Description: 3D stack through the IFT of a *tbx1*:EGFP/Isl1 double-stained embryo. Double positive cells are marked in dark blue (1), Isl1 single positive cells in light blue (2). Representative embryo as shown in Figure 2I-P (n=11 total number of analyzed embryos)

File Name: Supplementary Movie 3

Description: Lightsheet imaging of the cardiopharyngeal field of a *tbx1*:EGFP;*drl*:mCherry embryo. Maximum intensity projection with the dorsal view of the anterior region of a *tbx1*:EGFP;*drl*:mCherry transgenic, anterior to the top right corner; *tbx1*:EGFP depicted in green, *drl*:mCherry in magenta. Cardiac development was followed from 14 ss to 23 hpf, n=3. Movie stacks were acquired approximately every 7.5 min. Frame rates 7 fps. 1 sec in the movie corresponds approximately to 52.5 min in development.

File Name: Supplementary Movie 4

Description: Animated 3D segmentation of the *tbx1* reporter-expressing sheath at the base of the heart tube. Rotation of the 3D segmentation of still from Supplementary Movie 2 shown in Figure 3J-N revealing a *tbx1* reporter-expressing sheath of cells at the base of the forming heart tube and engulfing the *drl* reporter-expressing endocardium at 22-23 hpf.

File Name: Supplementary Movie 5

Description: Lightsheet-imaging of the developing heart tube of a *tbx1*:EGFP;*myl7*:DsRed2 embryo Maximum intensity projection with the dorsal view of the anterior region of a *tbx1*:EGFP;*myl7*:DsRed2 transgenic, anterior to the top; *tbx1*:EGFP depicted in green, *myl7*:DsRed2 in magenta. Heart tube development was followed from 18 ss to 30 hpf, n=2. Movie stacks were acquired approximately every 2.8 min. Frame rates 10 fps. 1 sec in the movie corresponds approximately to 28 min in development.

File Name: Supplementary Movie 6

Description: Time-lapse of a high-speed SPIM-imaged beating heart in a *tbx1*:EGFP;*myl7*:DsRed2 transgenic Heart development was followed from 28-52 hpf, all time points are synchronized and shown at contraction phase 27, heart imaged from right side of the embryo, lateral view, anterior to the top, ventricle to the upper left, atrium to the lower right; n=1. *tbx1*:EGFP is depicted in cyan, *myl7*:DsRed2 in red. Frame rates 7 fps. 1 sec in the movie corresponds approximately to 144 min in development.

File Name: Supplementary Movie 7

Description: *tbx1*:EGFP-expression in a beating heart at 28 hpf reconstructed from highspeed SPIM-imaging Volume rendering showing a cardiac cycle in slow motion in a 28 hpf embryo heart as imaged in Supplementary Movie 5. Only EGFP expression is shown to highlight that all *tbx1* reporter-expressing cardiac cells move during cardiac contraction.

File Name: Supplementary Movie 8

Description: SU5402 pulse-treated embryos (14 ss – 22 hpf) do not develop a BA as seen by *tbx1*:EGFP expression Rotation of the embryo shown in Figure 7J-L showing that the *tbx1*:EGFP+/*myl7*:dsRed- cannot be observed from any angle, n=4/8.

File Name: Supplementary Movie 9

Description: Embryos continuously treated with SU5402 from 14 ss do not develop a BA as seen by *tbx1*:EGFP expression Rotation of the embryo shown in Figure 7M-O showing that the *tbx1*:EGFP+/*myl7*:dsRed- cannot be observed from any angle n=3/3.
